# Induced Pluripotency and Gene Editing in Disease Modelling: Perspectives and Challenges

**DOI:** 10.3390/ijms161226119

**Published:** 2015-12-02

**Authors:** Yu Fen Samantha Seah, Chadi A. EL Farran, Tushar Warrier, Jian Xu, Yuin-Han Loh

**Affiliations:** 1 Epigenetics and Cell Fates Laboratory, A*STAR Institute of Molecular and Cell Biology, 61 Biopolis Drive Proteos, Singapore 138673, Singapore; yfseah@imcb.a-star.edu.sg (Y.F.S.S.); chadi@u.nus.edu (C.A.E.F.); e0011350@u.nus.edu (T.W.); 2 Department of Biological Sciences, National University of Singapore, Singapore 117543; dbsxj@nus.edu.sg; 3 NUS Centre for BioImaging Sciences, National University of Singapore, Singapore 117543

**Keywords:** iPSCs, CRISPR, gene editing, disease modeling

## Abstract

Embryonic stem cells (ESCs) are chiefly characterized by their ability to self-renew and to differentiate into any cell type derived from the three main germ layers. It was demonstrated that somatic cells could be reprogrammed to form induced pluripotent stem cells (iPSCs) via various strategies. Gene editing is a technique that can be used to make targeted changes in the genome, and the efficiency of this process has been significantly enhanced by recent advancements. The use of engineered endonucleases, such as homing endonucleases, zinc finger nucleases (ZFNs), transcription activator-like effector nucleases (TALENs) and Cas9 of the CRISPR system, has significantly enhanced the efficiency of gene editing. The combination of somatic cell reprogramming with gene editing enables us to model human diseases *in vitro*, in a manner considered superior to animal disease models. In this review, we discuss the various strategies of reprogramming and gene targeting with an emphasis on the current advancements and challenges of using these techniques to model human diseases.

## 1. Introduction

In 1963, Ernest Armstrong McCulloch and James Edgar Till demonstrated the presence of self-renewing cells in mouse bone marrow [1] and, in 1981, Martin Evans, Matthew Kaufman, and Gail R. Martin were able to derive embryonic stem cells (ESCs) from mouse embryos [2]. ESCs are characterized by their ability to self-renew indefinitely, as long as their genetic expression profile favors self-renewal [3]. In addition, they are pluripotent, namely they are able to differentiate into nearly any of the cell types derived from the three main germ layers that comprise an organism [4]. Due to these characteristics, ESCs are thought to be promising in the search for a cure for degenerative diseases [5].

In 2006, Kazutoshi Takahashi and Shinya Yamanaka successfully generated induced pluripotent stem cells (iPSCs) from mice fibroblast cultures by the addition of a few defined transcription factors [6]. Since then, many strategies for generating safer and more stable iPSCs from a variety of somatic cell types have been developed [7].

The discovery that somatic cells can be reprogrammed to form iPSCs, which share similar characteristics with ESCs, has expanded the prospect for development of cellular therapies for degenerative diseases [8]. iPSCs face less ethical controversy as compared to ESCs, and as patient-specific iPSCs can be generated from patients of most genotypes, it is easier to model a wide range of diseases with iPSCs.

Disease modeling is the process in which one generates a representation of a disease, in order to further understand disease pathology and etiology, and to aid in the development of diagnostics and therapy. This ties in with drug discovery, as accurate disease models can then be utilized for drug testing.

Once iPSCs are generated, gene editing can be utilized to further understand the genetic causes of the disease, via both the introduction of new mutations and the correction of suspected disease-causing mutations. The efficiency of gene editing has been enhanced significantly by the use of bioengineered endonucleases such as zinc finger nucleases (ZFNs) [9], homing endonucleases [10], transcription activator-like effectors (TALEs) [11] and the CRISPR/Cas system [12]. These introduce a double strand break at specific locations in the genome, and the action of the various DNA repair pathways can then generate changes in DNA sequence.

In this review, we examine the process of disease modeling utilising iPSCs, with an emphasis on the advances made in recent years, such as advances in reprogramming and gene editing, while considering the challenges that persist.

## 2. Disease Modeling

The process of utilising iPSCs for disease modeling is as follows: cells are obtained from healthy or diseased individuals and reprogrammed, before they are differentiated into a specific cell type for further studies. Additionally, gene editing may be carried out before differentiation, and this enables the generation of isogenic iPSC lines that differ only at specific loci, such that the effect of that single variation can be studied in detail [13]. This review will discuss the process of reprogramming and gene editing, before illustrating how these may be utilized in the generation of iPSC disease models.

## 3. Cellular Reprogramming

Cell identity is regulated by the epigenetic state of the cell, namely by modifications in DNA such as DNA methylation and histone modifications [14]. Oct4, Nanog and Sox2 are transcription factors that are crucial in maintaining the pluripotency and self-renewal of ESCs. The expression of these transcription factors lead to the suppression of genes associated with differentiation and the expression of genes that favor pluripotency [15]. This led to the belief that the induction of expression of these genes in somatic cells could cause cells to revert to a pluripotent state.

In 1962, Gurdon showed that differentiated nuclei could be reprogrammed to the pluripotent state, when he successfully generated animals by the transplantation of somatic nuclei into enucleated eggs [16], for which he won the Nobel Prize for Physiology and Medicine in 2012. Yamanaka shared this Nobel Prize, as in 2006, Takahashi and Yamanaka were able to convert mouse somatic fibroblasts to iPSCs by inducing the overexpression of four transcription factors, namely Oct4, Sox2, Klf4 and c-Myc (now known as Yamanaka factors). It came as quite a surprise that Nanog was not required for reprogramming [6]. Retroviral vectors were used to induce the ectopic expression of the four defined transcription factors that initiated events that reactivated genes associated with pluripotency. These reprogrammed cells were, like ESCs, found to be able to contribute to the germ-line in chimeric mice [17], and were capable of forming teratomas containing tissues derived from the three main germ layers [8]. Subsequently, it was also shown that the overexpression of other combinations of transcription factors, such as combinations including Nanog or Lin28, could trigger cellular reprogramming [18].

### 3.1. Transgene-Based Cellular Reprogramming Methods

Transgene-based cellular reprogramming methods are those that involve the induction of ectopic expression of defined transcription factors that leads to reprogramming in somatic cells, by integration of the genes encoding these transcription factors into the host genome. Takahashi and Yamanaka utilized retroviral vectors containing Oct4, Sox2, Klf4 and c-Myc, such that these genes were overexpressed, to reprogram mouse fibroblasts to iPSCs [6]. Similarly, Yu *et al.* [18] were able to generate iPSCs from both mouse and human fibroblasts by inducing the overexpression of Oct4, Sox2, Nanog, and Lin28, with the use of lentiviral vectors. iPSCs can also be generated via the use of an inducible lentiviral system, where iPS cell clones are differentiated into fibroblast-like cells, that can be induced to express reprogramming factors, to enable secondary reprogramming [19].

Another approach for transgene-mediated reprogramming is by the use of integrating non-viral inducible plasmid vectors. For example, Merkl *et al.* [20] used a doxycycline-inducible plasmid vector containing murine Oct4, Sox2, c-Myc and Klf4 to reprogram rat fibroblasts.

It was also found that the introduction of certain small molecules in combination with reprogramming factors could enhance reprogramming efficiency. The compound E-616452 (RepSox) was found to be able to replace Sox2 in the reprogramming of mouse embryonic fibroblasts (MEFs). RepSox acts by inhibiting the transforming growth factor-β (TGF-β), thus upregulating Nanog [21]. Kenpaullone, a GSK3 inhibitor, is another compound that enhanced the reprogramming of MEFs by complementing and thus, replacing Klf4 [22]. In addition, Lin *et al.* [23] demonstrated that when Yamanaka factors were combined with SB431542, an Alk5 inhibitor, PD0325901, a MEK inhibitor and thiazovivin, a 200-fold increase in reprogramming efficiency could be attained.

A variety of studies have also illustrated that certain small molecules are able to replace some of the Yamanaka factors in reprogramming. For example, compounds such as A-83-01, PD0325901, PS48, 0.25 mM sodium butyrate [24], Vitamin C [25], BIX-01294, BayK8644 [26], and valproic acid (VPA) [27] are able to either replace factors assumed to be crucial for reprogramming, or increase reprogramming efficiency [28]. In addition, Hou *et al.* [29] demonstrated that seven small-molecule compounds were able to reprogram mouse somatic cells in the absence of the expression of exogenous transcription factors.

Due to the ease of utilising transgene-based reprogramming, these methods remain the most widely used strategies in reprogramming. However, as the site of viral integration is usually random, viral-mediated reprogramming carries the risk of insertional inactivation of a vital gene or perturbation of endogenous gene expression [8]. Another problem associated with this type of cellular reprogramming is low reprogramming efficiency [8].

### 3.2. Transgene-Free Cellular Reprogramming Methods

Due to the risks and limitations associated with viral-mediated cellular reprogramming methods, several other methods for generating iPSCs have been developed. As mentioned above, it is now possible to reprogram mouse somatic cells with small-molecule compounds in the absence of exogenous transcription factors [29]. The ability to generate human iPSCs utilising small-molecule compounds alone is a highly desired goal as small-molecule reprogramming has a smaller risk of perturbing endogenous gene sequences or expression [28].

Alternatively, iPSCs can be generated using non-integrating plasmid vectors. The transient co-transfection of plasmids encoding the Yamanaka factors enabled the generation of iPSCs from mouse embryonic fibroblasts [30].

Non-integrating viral mediated cellular reprogramming can be achieved by using RNA viruses that do not integrate their genes into the host genome. In one approach, Yu *et al.* [31] cloned six reprogramming factors (Oct4, Sox2, Nanog, LIN28, c-Myc and Klf4) into an oriP/EBNA1 (Epstein-Barr nuclear antigen-1) based episomal vector and, thus, were able to reprogram human fibroblasts into iPSCs. In addition, multiple labs have also made use of Sendai viruses to reprogram somatic cells such as human fibroblasts [32] and human peripheral blood cells [33]. Similarly, non-integrating DNA adenoviral vectors encoding Yamanaka factors have been successfully used to reprogram MEFs, mouse liver cells [34] and human embryonic fibroblasts [35].

Transgene-free cellular reprogramming can also be achieved by utilising modified lentiviral vectors in which the vectors can be excised from the genomes of the generated iPSCs. For example, Chang *et al.* [36] successfully generated iPSCs from dermal fibroblasts by using a polycistronic lentiviral vector that encoded the reprogramming factors Oct4, Sox2, and Klf4. This lentiviral vector contained a loxP site in the 3′-LTR region, such that the vector could be deleted upon the expression of Cre recombinase. Similarly, Sommer *et al.* [37] successfully generated iPSCs from peripheral blood mononuclear cells by using a single excisable polycistronic lentiviral Stem Cell Cassette (STEMCCA) that encoded Yamanaka factors.

Similarly, modified transposons can be utilized for transgene-free cellular reprogramming. Yusa *et al.* [38] used a piggyBAC-derived transposon system carrying 2A peptide-linked reprogramming factors for reprogramming, which was subsequently removed by the re-expression of transposase. The piggyBAC system excises without a footprint, such that the impact on the genome is minimized.

Another approach for transgene-free mediated reprogramming is the use of synthetic mRNA encoding Yamanaka factors. These can be introduced into cells via complexing with cationic vehicles [39] or by electroporation [40].

In addition, transgene-free cellular reprogramming can be achieved by the use of recombinant proteins, such as Yamanaka factors fused to poly-arginine domains. Proteins containing such poly-arginine domains are able to easily cross cell membranes, and mouse embryonic fibroblasts (MEFs) [41] and human newborn fibroblasts (HNFs) [42] were successfully reprogrammed using this approach. Reprogramming factors can also be transfected into cells by magnet-based nanofection, where proteins are conjugated to non-viral magnetic nanoparticles, enabling their easy transfection into cells via magnetic force [43].

Micro RNAs (miRNAs) can also be used to generate iPSCs, and this does not involve the utilization of any of the reprogramming factors that are commonly used. Anokye-Danso *et al.* [44] used a lentiviral vector to induce the expression of mouse miR302/367 in MEFs and human fibroblasts. Here, reprogramming was successful with a higher efficiency when compared with reprogramming using Yamanaka factors.

These transgene-free methods eliminate the risk of random integration, and thus may be preferred, but these methods are often tedious and tend to have lower efficiencies.

**Figure 1:**
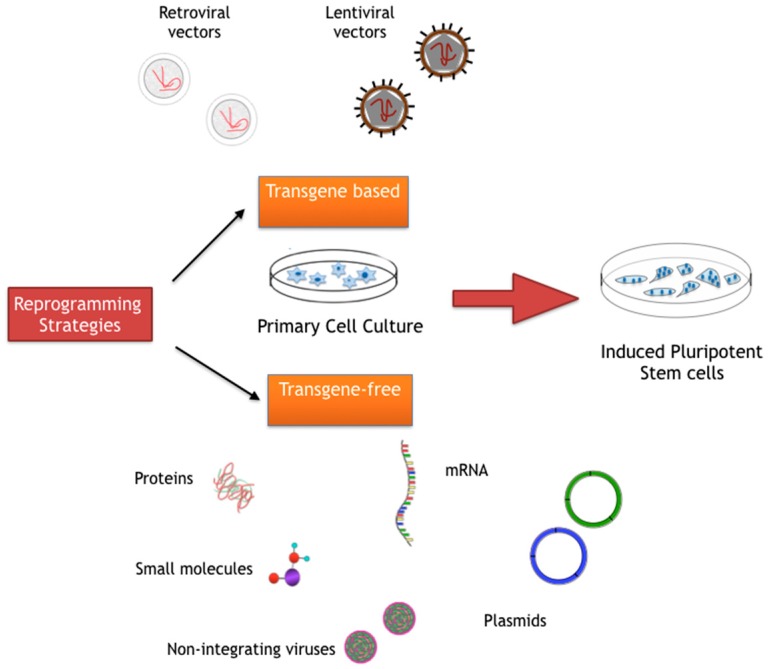
Schematic displaying the various reprogramming strategies used in generation of induced pluripotent stem cells (iPSCs).

The methods discussed above have been utilized for reprogramming various cell types into iPSCs (Figure 1, Table 1). These iPSCs can then be directly differentiated into the cell type of interest to model diseases, or have their genome modified by gene editing before differentiation, as discussed below.

**Table 1:** Transgene-based and transgene-free methods of reprogramming. (C—CHIR, F—FSK, K—Klf4, L—Lin28, M—c-Myc, N—Nanog, O—Oct4, S—Sox2, T—TTNPB, V—VPA, Z—DZNep and 6—616452). (All efficiencies for reprogramming are indicated as the percentage for the number of induced pluripotent stem cell (iPSC) colonies as compared to the original number of seeded cells).

Method Type	Method	Factors/Other Agents	Cell Type	References	Efficiency
Transgene-based	Retroviral	OSKM	Mouse fibroblasts	[6]	0.02%
	Retroviral + small molecules	OSKM + SB431542 + PD0325901	Human fibroblasts	[23]	~1%
	Lentiviral	OSNL	Mouse fibroblasts, human fibroblasts	[18]	0.0095%
	Inducible lentiviral	OSKMN	Human fibroblasts, keratinocytes	[19]	1%–3%
	Integrating, non-viral inducible plasmid vectors	OSKM	Rat fibroblasts	[20]	0.0027%–0.0078%
Transgene-free	Small molecules	C6FZ or VC6TFZ	Mouse fibroblasts	[29]	0.2%
	Episomal plasmids	OSKM	Mouse fibroblasts	[17]	~0.1%
	Sendai viruses	OSKM	Human fibroblasts	[32]	~1%
	Non-integrating DNA adenoviral	OSKM	Tail tip fibroblasts, hepatocytes, fatal liver cells	[34]	0.0001%–0.001%
	Excisable lentiviral	OSK	Mouse fibroblasts, human fibroblasts	[36]	Not reported
	PiggyBAC transposon	OSKM or OSKML	Mouse fibroblasts	[38]	~1%
	Synthetic mRNA	OSKM	Human fibroblasts	[39]	2%
	Polyarginine-tagged polypeptides	OSKM	Human fibroblasts	[42]	0.001%
	Magnet-based nanofection of polypeptides	OSKM	Mouse fibroblasts	[43]	0.001%–0.003%
	MicroRNAs (lentiviral)	miR302/367	Mouse fibroblasts, human fibroblasts	[44]	Up to 10%

### 3.3. Cell Types from Which Induced Pluripotent Stem Cells (iPSCs) Have Been Derived

iPSCs have been generated from a plethora of somatic cell types for both mice and humans. These include but are not limited to B-lymphocytes [45], neural progenitor cells (NPCs) [46], hepatocytes and gastric epithelial cells [21] from mice as well as adipose-derived stem cells [47], keratinocytes [48], peripheral blood T-cells [49,50], hair follicle cells [51], amniotic fluid cells [52] and astrocytes [53] from humans. In addition, Park *et al.* [54] have shown that iPSCs could be generated from fetal, neonatal and adult human primary cells, including dermal fibroblasts isolated from healthy subjects.

## 4. Gene Targeting and Disease Modeling

Gene editing is the process by which one makes targeted changes to genomic DNA sequences, such as insertions, deletions, point mutations or translocations. This is done by the targeted generation of double strand breaks (DSBs), which triggers the action of various DNA repair pathways, such as homology-directed repair (HDR) or non-homologous end joining (NHEJ). The imprecise NHEJ pathway results in insertion or deletion mutations (INDELs), as the blunt ends are joined together, frequently resulting in frameshift mutations or premature stop codons, and even in knockouts [55,56]. In contrast, if cleavage takes place in the presence of DNA that is partially homologous to the cleaved strand, the exogenous DNA may be incorporated into the genome by HDR [9,57]. This makes such cleavage useful in the introduction of foreign DNA into the eukaryotic genome, where HDR can enable the specific addition of exogenous protein-coding sequences [58].

Sequence-specific DNA-binding proteins such as homing nucleases, zinc finger proteins (ZFPs), transcription activator-like effectors (TALEs) and Cas9 of the CRISPR/Cas system have been adapted to introduce DSBs in a sequence-specific manner, to trigger HDR or NHEJ. The drawbacks of homing nucleases [59], ZFNs [60,61] and TALENS [62] (Table 2), along with the ease of re-targeting in the CRISPR/Cas9 system, have led to the widespread adoption of the CRISPR/Cas9 system for gene editing.

**Table 2:** Summary of different programmable nuclease systems available and the systems from which they were adapted.

System	Endogenous System	Advantages	Disadvantages
Homing endonucleases	Restriction enzymes with large restriction sites, encoded by mobile genetic elements [63]	□ Small protein size, potentially easier to introduce into cells *ex vivo* and *in vivo* [64]	□ Re-targeting requires protein engineering [65] □ No obvious correlation between amino acid sequence and DNA recognition sites [59]
Zinc finger nucleases (ZFNs)	Zinc finger domain found in many eukaryotic transcription factors [66,67]	□ Small protein size, potentially easier to introduce into cells *ex vivo* and *in vivo* □ Likely low immunogenicity effects, as zinc fingers are based on human protein scaffolds	□ Re-targeting requires protein engineering [68] □ Expensive to produce [61] □ Lack of correspondence between amino acid sequence and DNA recognition sites [68] □ Context-dependent specificity [60,66] □ Off target effects [61]
Transcriptional activator-like effector nucleases (TALENs)	TAL-effector proteins from *Xanthomonas*, a plant pathogen [69]	□ Lack of context dependence, assembly more straightforward [70]	□ Complex molecular cloning (Golden Gate Assembly) required to produce [62] □ Large protein size, potentially difficult to introduce into cells *ex vivo* and *in vivo* [71]
CRISPR/Cas9	Prokaryotic adaptive immune system [72]	□ High multiplexing efficiency [73] □ Easy to re-target (cloning and oligo synthesis) [74] □ Depends on predictable Watson-Crick base-pairing	□ Off-target effects [75]

## 5. CRISPR/Cas9 System and Its Potential in Disease Modeling

CRISPR/Cas systems were the first type of the prokaryotic adaptive immune system discovered, and are perhaps the most complex. Scientists first noticed CRISPR repeats in 1987 [76], but their function was not elucidated until 2005 [77]. It was later experimentally shown that the Type II CRISPR system acts as an adaptive immune system, to protect the prokaryotic genome against mobile genetic elements [72].

Of the different CRISPR/Cas systems, eukaryotic genome engineering has primarily utilised the Type II system from *Streptococcus pyogenes* [12,78]. The Type II system was selected as it is the most compact; the Cas9 protein and RNA constructs are sufficient to cleave target DNA [79], as compared to multiple Cas proteins needed in the Type I and Type III CRISPR/Cas systems.

The Type II CRISPR system requires two pieces of RNA to function, namely crRNA, which base pairs with target DNA, and tracrRNA, which triggers pre-crRNA processing and cleavage of the target by Cas9. These two RNA strands can be engineered into a single chimeric RNA, the single guide RNA (sgRNA), which functions as efficiently as the two endogenous RNAs [74]. This increases the ease of manipulation, as only two components (Cas9 and sgRNA) must be introduced into eukaryotic cells for the CRISPR system to be utilised.

However, a key problem in utilization of the CRISPR/Cas system is the high frequency of off-target effects [75]. Various strategies have been used to overcome this, such as increasing the length of the sequence recognized, by incorporating cooperativity into the functioning of the system. This has been done by fusing deactivated Cas9 proteins (dCas9) to FokI nucleases in a manner similar to that seen in the construction of ZFNs and TALENs. Here, two different sgRNAs binding at adjacent locations in the genome are required for cleavage to take place, reducing off-target effects, as it is unlikely that both sgRNAs will bind in close proximity outside the intended target [69]. Alternatively, Cas9 nickases can be made, where one of the two Cas9 nuclease domains is inactivated. The D10A mutation inactivates the RuvC-like domain, while the H840A mutation inactivates the HNH domain [80]. The introduction of two different sgRNAs targeting slightly different regions on opposite strands of the target results in two staggered nicks created by the paired nickases that results in a DSB [81].

## 6. Gene Editing in Disease Modeling

Such gene editing technologies enable the editing of a genome in a specific and targeted way. This could enable the generation of mutations in specific genomic locations, to create disease models that enable further study of disease pathology, or to investigate the effects of specific loci or mutations on the disease phenotype [13].

Specific corrections could be made in iPSCs of diseased individuals, to elucidate the effect of the specific mutant allele and to act as a proof-of-principle for gene therapy. In contrast, disease-associated mutations can be introduced into a healthy cell background, to elucidate the effect of those mutations [13].

ZFNs have been utilized for the creation of disease models. For example, Meyer *et al.* [82] used ZFNs to introduce missense and silent mutations into the *Rab38* gene, which encodes for a small GTPase that regulates intracellular vesicle trafficking. The introduction of these ZFNs in one-cell mouse embryos was used to generate disease-related mutants containing single nucleotide or codon replacements.

The CRISPR/Cas system has also been utilized in understanding disease in iPSC disease models. Soldner *et al.* [83] modeled a familial form of Parkinson’s disease (PD) by the generation of iPSCs from individuals carrying the A53T mutation in α-synuclein (*SNCA*) and corrected the mutation by gene editing. In addition, gene editing was used to introduce the A53T or E46K mutation in *SNCA* in wild-type hiPSCs, thus enabling the creation of iPSCs that were bi-allelic for the mutation. This enabled the study of mutant α-synuclein in the absence of wild-type α-synuclein, which could shed light on PD pathogenesis [83].

In addition, in order to study the role of the N996I *KCNH2* mutation in long-QT syndrome (LQTS), Bellin *et al.* [84] created iPSCs from individuals carrying the mutation and corrected the mutation via gene editing, while separately introducing the point mutation into hESCs. The iPSCs were differentiated into cardiomyocytes, with parameters such as the current conducted and AP duration studied in order to illustrate that the N996I *KCNH2* mutation was the main cause of the LQTS phenotype.

## 7. Advantages of iPSCs for Disease Modeling

The ability of iPSCs to be renewed indefinitely enables the generation of a large number of cells, aiding further study and large-scale screening, while the ability to generate patient-specific iPSCs enables the study of the genetic causes of diseases, as well as the generation of models of complex diseases for which the genetic causes may not have been fully elucidated. In addition, the simultaneous study of different patient-specific iPSC lines can aid in our understanding of how various disease-associated loci may interact to cause a phenotype. This could enhance disease diagnosis, and make drug screening personalized and more reliable [85].

In fact, iPSCs have been utilized as a diagnostic tool in a patient with long QT syndrome caused by a novel mutation [86]. Here, iPSCs were generated from the patient and differentiated into cardiomyocytes before being characterized by electrophysiological analysis and the introduction of specific drugs known to affect QT length. This illustrates that disease-specific iPSCs are able to recapitulate disease phenotypes, and could aid in both diagnosis and drug testing.

The utilization of disease modeling via iPSCs provides many advantages over the use of animal disease models. For example, human disease models will more accurately represent the physiology of human cells and systems as compared to animal models. As such, disease modeling via iPSCs will reflect both drug efficacy and toxicity more accurately. Currently, many drugs are initially tested in animals, such as mice, but this process may yield both false positives and negatives. For example, false positives include drugs that appear to alleviate the disease phenotype in mice, but that do not benefit humans. This was seen for creatine in the treatment of ALS (Amyotrophic Lateral Sclerosis), where it prolonged lifespan and maintained motor neuron function in mice models, but failed to yield any apparent benefit in human clinical trials [87]. In addition, drug toxicity differs between different animals, such that animal models may not be appropriate for testing drug toxicity [88]. While most clinical trials are expensive and time-consuming, pre-testing in more accurate iPSC disease models may be able to reduce cost and time [89].

## 8. Requirements of iPSCs in Disease Modeling

Before one is able to use generated iPSCs for disease modeling, the iPSCs should be screened for any mutations that could have occurred due to the reprogramming event [90]. The DNA methylation patterns of these iPSCs should also be examined to avoid any incomplete demethylation of crucial genes [91]. Also, there should be no change in the allelic copy number variation [92] or any abnormality of X chromosome inactivation [93]. The presence of any of these abnormalities can lead to changes in the phenotype of the iPSCs and of the cells generated from them.

It is important to keep in mind that iPSCs carry epigenetic memory from their somatic cells of origin, which plays a role in the tendency of iPSCs to differentiate into specific cell types. Thus, the somatic cell source must be compatible with the cell type of the desired disease model. For example, Moad *et al.* [94] showed that iPSCs generated from prostate and urinary tract cells had better efficiency of differentiation into prostate and urinary tract cells as compared to iPSCs derived from skin fibroblasts. This illustrates the epigenetic differences between cell types, and illustrates that the origin of cell type plays a key role in efficiency of targeted differentiation.

## 9. Examples of Disease Models

**Figure 2:**
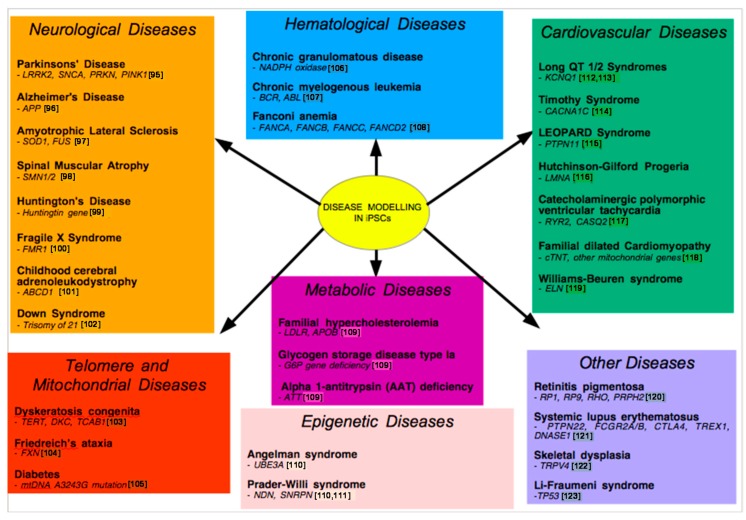
Schematic chart displaying the diseases that have been extensively modeled using gene editing and induced pluripotency [95,96,97,98,99,100,101,102,103,104,105,106,107,108,109,110,111,112,113,114,115,116,117,118,119,120,121,122,123].

iPSC-based disease models have been created for several diseases affecting different systems in the human body, including both Mendelian and complex diseases. This illustrates that iPSC-based disease models can be generated even if the exact genetic cause of the disease is not well understood (Figure 2). In addition, directed differentiation processes must exist, in order to differentiate the iPSCs into the cell type of interest [89].

### 9.1. Neurological Disease Models

Some of the first diseases to be modeled using iPSCs were neurological diseases, as neurons have been sufficiently well-studied such that good differentiation protocols exist, and as primary human neurons are extremely inaccessible, making an iPSC-based method desirable [13]. In addition, in neurodegenerative diseases such as Parkinson’s disease (PD), clinical manifestation usually indicates that certain neurons, in this case, dopaminergic neurons of substantia nigra, have already been lost, thus making the disease difficult to study by the isolation of primary cells of interest [124].

Disease models of Parkinson’s disease have been created by generating iPSCs from patients carrying the G2019S mutation in the *Leucine-Rich Repeat Kinase-2* (*LRRK2*) gene, the most common mutation in Parkinson’s disease. These iPSCs were differentiated into dopaminergic (DA) neurons, which accurately recapitulated the phenotype of Parkinson’s disease [95].

In addition, disease models via iPSCs have been created for Alzheimer’s disease [96], amyotrophic lateral sclerosis (ALS) [97], spinal muscular atrophy (SMA) [98], Huntington’s disease [99], fragile X-syndrome [100] and childhood cerebral adrenoleukodystrophy (CCALD) [101] amongst others. Differentiation of iPSCs into the cell types of interest enables further study of disease etiology and pathology.

In 2013, Lu *et al.* [102] successfully modeled the neurogenesis impairment in Down Syndrome by generating iPSCs from Trisomy 21 amniotic fluid cells. These generated iPSCs maintained three copies of chromosome 21. The level of amyloid precursor protein was significantly increased in the neuronal progenitor cells derived from the generated iPSCs, shedding light on the mechanism by which Trisomy 21 results in neurological defects.

### 9.2. Metabolic Disease Modeling

In 2010, Rashid *et al.* [109] demonstrated that metabolic diseases of the liver could be modeled using iPSC-based disease modeling. Dermal fibroblasts from patients with various inherited metabolic diseases of the liver were used to generate iPSCs that were subsequently differentiated into hepatocytes for modeling. The diseases that could be modeled using this method were familial hypercholesterolemia (FH), glycogen storage disease type Ia (GSDIa) and Alpha 1-antitrypsin (AAT) deficiency.

Other metabolic diseases of the liver such as tyrosinemia, glycogen storage disease, progressive familial hereditary cholestasis, and Crigler-Najjar syndrome have been successfully modeled [125].

### 9.3. Modeling Drug Metabolism

The key aspects of drug toxicity are cardiotoxicity and hepatotoxicity, and the differentiation of iPSCs into cardiac cells and hepatocytes is thus useful for testing new drugs. This can be done for iPSCs from healthy individuals—simply to study the effect of genetic variation on drug metabolism [89] or for individuals with inherited metabolic disorders in order to study their responses to drugs. This was done for individuals with alpha-1 antitrypsin (AAT) deficiency and it enabled the identification of five drugs that could alleviate the disease phenotype [126]. In addition, the ability to generate a plethora of cell types from iPSCs enables the evaluation of drug toxicity against many cell types, giving hope to a future of personalized medicine [127].

### 9.4. Cardiovascular Disease Models

Moretti *et al.* [112] have successfully generated iPSCs from patients suffering long QT 1 syndrome, caused by a missense mutation in the *KCNQ1* gene. The iPSCs generated were differentiated to cardiomyocytes that recapitulated the phenotype of the disease, including increased depolarization of cardiomyocytes.

In another study, Itzhaki *et al.* [113] generated iPSCs from patients suffering from long QT 2 syndrome, and these carried the monogenic A614V missense mutation in the *KCNH2* gene. The differentiated cardiomyocytes recapitulated the disease phenotype and showed an increase in their depolarization.

In 2011, Yazawa *et al.* [114] modeled Timothy syndrome successfully. Other cardiovascular diseases that have been successfully modeled include but are not limited to LEOPARD syndrome [115], Hutchinson-Gilford progeria syndrome (HGPS) [116], catecholaminergic polymorphic ventricular tachycardia [117], familial dilated cardiomyopathy [118] and Williams-Beuren syndrome [119].

### 9.5. Telomere Disease Models

In 2011, Batista *et al.* [103] generated iPSCs from dyskeratosis congenita patients that demonstrated telomere shortening and loss of telomere self-renewal. The iPSCs were found to harbour the precise biochemical defects which are characteristic of the disease. This illustrates that iPSCs themselves can be used to study mechanisms of human stem cell diseases.

### 9.6. Other Disease Models

In addition, various diseases of different tissues have been successfully modeled. These include hematological diseases like various myeloproliferative disorders [128], chronic granulomatous disease [106], chronic myelogenous leukemia [107] and Fanconi anemia [108]. Mitochondrial diseases like Friedreich’s ataxia [104] and diabetes caused by the mtDNA A3243G mutation [105] have also been modeled. Retinitis pigmentosa, the leading cause of blindness in industrial countries [120], systemic lupus erythematosus [121] and skeletal dysplasia [122] have also been modeled. The plethora of examples illustrate that iPSCs enable the modeling of many different diseases that affect various different tissues.

### 9.7. Modeling Epigenetic Diseases

Angelman and Prader-Willi syndrome were also successfully modeled. The generated iPSCs had the DNA methylation patterns characteristic of each disease and the differentiated neurons recapitulated the phenotypes of both diseases [110,111]. This is particularly interesting as Angelman and Prader-Willi syndrome are caused by inappropriate imprinting, and the successful modeling of these diseases indicate that epigenetic diseases can similarly be modeled by iPSCs as long as genomic imprinting marks are not disturbed [111].

### 9.8. Modeling Infectious Diseases

Disease modeling using iPSCs is not restricted to diseases with genetic causes, in fact, patient-specific iPSC-derived cells can be utilized as platforms to analyze host–pathogen interactions, where the cells are infected with a virus and studied. iPSC-derived cells are more accurate representatives of human physiology as compared to animal models [129]. iPSC-based disease modeling is particularly useful for studying viruses that are highly species-specific, or that can only grow in a restricted number of cell types, specifically those that may be hard to isolate and culture [129]. For example, herpes simplex virus (HSV) and varicella zoster virus (VZV) have tropism for neural cells and establish latency in sensory neurons, and these cell types can be generated from iPSCs to enable further study of these viruses. In addition, patient-specific iPSCs can be used to further understand the genetic bases of viral infections, as have been done for HSV encephalitis [130] and severe influenza [131]. Genome editing has also been utilized in iPSCs as a bid to confer antiviral resistance, as has been done for HIV, by generating anti-HIV iPSCs that contained CCR5 shRNA in combination with a chimeric human/rhesus TRIM5α molecule that could generate HIV-resistant macrophages [132].

### 9.9. Modeling Cancer

Cancer cell lines from human tumours have been immortalized and have been used to study cancer, but prolonged culture could result in these cell lines being less representative of primary tumours [133]. In addition, many of these cell lines represent a mature cancer stage, and are not helpful in modeling early cancer stages. As such, iPSCs have been generated from a number of cancer cell lines or cancer cells from biopsies, in the hope that these will enable the development of an *in vitro* model for carcinogenesis and recapitulation of cancer development. For example, the lack of a human cell model of the early stages of pancreatic ductal adenocarcinoma (PDAC) limited the study of PDAC development, but the generation of iPSCs from PDAC lines and injection of these iPSCs into mice enabled the study of PDAC progression [134].

In addition, iPSC lines have been generated from a family with Li-Fraumeni syndrome, a familial cancer syndrome linked to mutations in the TP53 tumor suppressor gene. These iPSCs were used to study the role of mutant p53 in the development of osteosarcomas in these patients, demonstrating how iPSCs can be utilized to study inherited cancer syndromes [123].

It is widely believed that pluripotency and oncogenic transformation are related processes [135,136], due to the presence of common characteristics, such as self-renewal, altered metabolism and expression of certain markers and stem cell genes [137]. iPSCs might thus be useful in understanding certain properties of cancer cells.

## 10. Limitations of iPSCs in Disease Modeling

Despite its application for various diseases, the use of iPSCs continues to be limited by various factors such as epigenetic memory, the absence of efficient differentiation protocols and non-genetic variability between cells. In addition, it is still difficult to study epigenetic diseases, as well as diseases that involve more than one cell type, diseases that are affected by the environment and adult onset diseases.

Although iPSCs share many similar characteristics with ESCs, such as the expression of stem cell markers such as SSEA-1 and alkaline phosphatase, the *in vitro* and *in vivo* differentiation into the three germ layers and the ability to form teratomas [6], recent studies have demonstrated that the DNA methylation pattern of iPSCs resembles that of their cells of origin, thus pushing iPSC differentiation towards the lineage of the cells of origin, as previously mentioned. This phenomenon has been termed epigenetic memory [138]. This affects the cell types that can be effectively generated, thus the cell type of origin and desired cell type must be carefully considered when planning such experiments.

The use of iPSCs is currently limited to cell types for which there are dependable and efficient differentiation protocols, and this might limit the cell types that could be modeled. Protocols do exist for well-studied cell types, such as neural cells, but may not be present for other cell types, such that the efficiency of iPSC differentiation into a specific cell lineage may not be sufficient to model certain diseases [139]. In addition, the lack of efficient and robust differentiation protocols may result in a heterogeneous mixture of cell types, which may not be useful if the aim is to model a disease in a single cell type. This might be overcome by the use of reporter genes placed under cell-specific promoters, to effectively select for cells of the cell type of interest [140].

Individual iPSC lines may exhibit highly variable properties independently of genetic background. This could be due to cellular changes resulting from reprogramming, particularly if transgene-based reprogramming is utilized, as transgenes may integrate into the genome and disrupt endogenous gene expression [141]. This could be overcome by utilising transgene-free reprogramming, and by adopting more stringent quality controls.

## 11. Diseases That Continue to Be Difficult to Model

Not all diseases are applicable for modeling using iPSCs. For example, diseases that are caused by changes in the epigenetic status of cells may not be effectively modeled by iPSCs as the epigenetic status of cells may change during cellular reprogramming [142]. A notable exception to this is mentioned above, namely the fact that Angelman and Prader-Willi syndrome can be modeled using iPSC-based disease modeling [111].

Modeling of diseases is made more complex by the fact that many diseases involve complex interactions between multiple cell types, such that studying a single cell type or a limited number of cell types independently will not be able to explain the pathology of the disease. For example, the pathogenesis of liver disease is affected by the complex interactions between various types of parenchymal and non-parenchymal cells, and studying a single cell type alone would be insufficient in understanding the disease [127]. Similarly, iPSC-based disease modeling cannot model the three-dimensional (3D) niche in which cells usually find themselves.

However, this may be overcome by the recent development in various 3D culture techniques, as well as the development in organoid-growing and tissue engineering techniques. This has been illustrated for a variety of organs. For example, iPSC-derived hepatic endoderm cells cultured with endothelial and mesenchymal cells form liver buds, and form functional human liver tissue upon transplantation into mice [143]. Similarly, iPSCs directed to undergo neural differentiation and cultured in a 3D system are able to form cerebral organoids. These organoids develop discrete brain regions, and can be subsequently used to model microcephaly [144]. 3D culture iPSC models of Alzheimer’s disease (AD) have also been generated, which appear to have a greater resemblance to actual AD pathology as compared to conventional 2D cultures [145]. In addition, human pluripotent stem cells (hPSCs) have been differentiated to generate 3D optic cups that could be used to model retinal diseases [146], and kidney-like structures that could be used to model renal diseases [147].

It is also difficult to model diseases that are affected by environmental factors. For example, alcohol-mediated liver damage is largely influenced by alcohol consumption, and would be difficult to model, especially if the cells were sourced from non-liver tissues [127].

Adult onset diseases are similarly difficult to model. iPSCs are characterized by the fact that they resemble ESCs, which are in a fetal-like stage of development. As such, they might not exhibit the phenotype of adult-onset diseases, such as Parkinson’s or Alzheimer’s disease. This could be overcome by artificially “ageing” cells, which has been done in iPSC-derived neurons by exposing cells to oxidative stress [95], by expressing progerin [148] or by excessively stimulating neurons with high concentrations of glutamate [149], which all induce age-dependent disease phenotypes (Table 3).

**Table 3:** Challenges in utilising iPSCs for disease modeling and their potential solutions.

Challenges	Potential Solutions
Epigenetic memory [138]	Test various cell types, and use late passage iPSCs
Lack of dependable and efficient differentiation protocols, which may result in the generation of a mixture of cell types [139]	Further research into and development of protocols utilise reporter genes to select for the cell type of interest [140]
Variable properties independent of genetic background, especially for transgene-based reprogramming	Utilise transgene-free reprogramming utilise more stringent quality controls
Modeling of diseases involving the complex interactions between multiple cell types, in a three-dimensional niche [127]	Advances in 3D culture techniques, organoid-growing techniques and tissue engineering strategies [143,144,146,147]
Modeling of diseases affected by environmental factors	Use cells cultured in bio-engineered niches and co-culture with primary cells in order to mimic *in vivo* tissue development
Modeling of adult onset diseases	For neurons, exposing cells to oxidative stress [95], progerin [148], or by excessively stimulating neurons with high concentrations of glutamate [149]

## 12. Concluding Remarks

iPSC-based disease modeling has been utilized for many diseases, supplementing more traditional animal disease models. The current techniques in cellular reprogramming have provided a suitable platform for modeling diseases and recapitulating their phenotypes. Transgene-free methods are especially useful, as these reduce the risk of unspecific alterations in the gene expression profile of the cells. Another breakthrough in cellular reprogramming has been the generation of iPSCs from blood and other samples. This breakthrough made obtaining patients’ cells easy and non-invasive. Samples from infants, children and elderly people can now be easily collected with little risk. The easy isolation of patient-specific iPSCs enables population-wide modeling of diseases, eventually contributing to personalized medicine.

**Figure 3:**
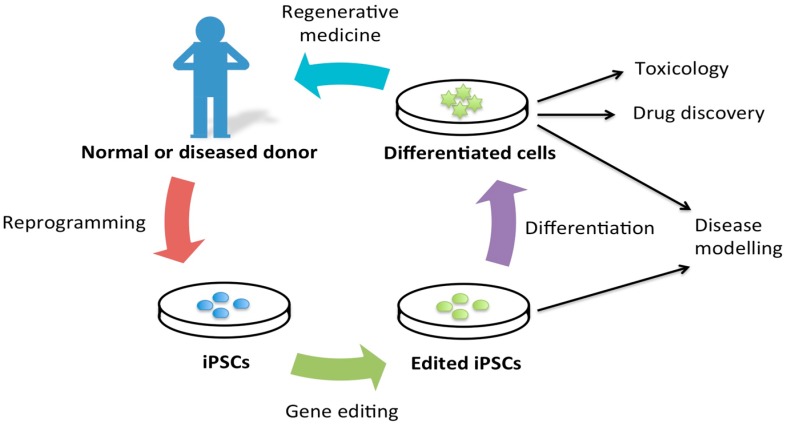
Schematic displaying how reprogramming and gene editing can contribute to disease modeling, toxicology, drug discovery, and potentially to regenerative medicine.

Gene editing via the use of ZFNs, TALENs and particularly via CRISPR enable us to study and model diseases with higher efficacy. Scientists can now edit the genomes of iPSCs derived from healthy or diseased individuals, to better understand the genetic causes of diseases (Figure 3).

However, there remain limitations to the use of iPSCs in disease modeling, which must be overcome. We continue to struggle to model complex diseases that are affected by the environment, or that involve the interaction between multiple cell types.

## References

[1] Siminovitch L., McCulloch E.A., Till J.E. (1963). The distribution of colony-forming cells among spleen colonies. J. Cell. Comp. Physiol..

[2] Martin G.R. (1981). Isolation of a pluripotent cell line from early mouse embryos cultured in medium conditioned by teratocarcinoma stem cells. Proc. Natl. Acad. Sci. USA.

[3] He S., Nakada D., Morrison S.J. (2009). Mechanisms of stem cell self-renewal. Annu. Rev. Cell Dev. Biol..

[4] Ulloa-Montoya F., Verfaillie C.M., Hu W.S. (2005). Culture systems for pluripotent stem cells. J. Biosci. Bioeng..

[5] Lindvall O., Kokaia Z. (2006). Stem cells for the treatment of neurological disorders. Nature.

[6] Takahashi K., Yamanaka S. (2006). Induction of pluripotent stem cells from mouse embryonic and adult fibroblast cultures by defined factors. Cell.

[7] Hanna J.H., Saha K., Jaenisch R. (2010). Pluripotency and cellular reprogramming: Facts, hypotheses, unresolved issues. Cell.

[8] Brock A., Goh H.T., Yang B., Lu Y., Li H., Loh Y.H. (2012). Cellular reprogramming: A new technology frontier in pharmaceutical research. Pharm. Res..

[9] Bibikova M., Beumer K., Trautman J.K., Carroll D. (2003). Enhancing gene targeting with designed zinc finger nucleases. Science.

[10] Grizot S., Smith J., Daboussi F., Prieto J., Redondo P., Merino N., Villate M., Thomas S., Lemaire L., Montoya G. (2009). Efficient targeting of a SCID gene by an engineered single-chain homing endonuclease. Nucleic Acids Res..

[11] Miller J.C., Tan S., Qiao G., Barlow K.A., Wang J., Xia D.F., Meng X., Paschon D.E., Leung E., Hinkley S.J. (2011). A TALE nuclease architecture for efficient genome editing. Nat. Biotechnol..

[12] Cong L., Ran F.A., Cox D., Lin S., Barretto R., Habib N., Hsu P.D., Wu X., Jiang W., Marraffini L.A. (2013). Multiplex genome engineering using CRISPR/Cas systems. Science.

[13] Sterneckert J.L., Reinhardt P., Schöler H.R. (2014). Investigating human disease using stem cell models. Nat. Rev. Genet..

[14] Goldberg A.D., Allis C.D., Bernstein E. (2007). Epigenetics: A landscape takes shape. Cell.

[15] Loh Y.H., Wu Q., Chew J.L., Vega V.B., Zhang W., Chen X., Bourque G., George J., Leong B., Liu J. (2006). The Oct4 and Nanog transcription network regulates pluripotency in mouse embryonic stem cells. Nat. Genet..

[16] Gurdon J.B. (1962). The transplantation of nuclei between two species of *Xenopus*. Dev. Biol..

[17] Okita K., Ichisaka T., Yamanaka S. (2007). Generation of germline-competent induced pluripotent stem cells. Nature.

[18] Yu J., Vodyanik M.A., Smuga-Otto K., Antosiewicz-Bourget J., Frane J.L., Tian S., Nie J., Jonsdottir G.A., Ruotti V., Stewart R. (2007). Induced pluripotent stem cell lines derived from human somatic cells. Science.

[19] Maherali N., Ahfeldt T., Rigamonti A., Utikal J., Cowan C., Hochedlinger K. (2008). A high-efficiency system for the generation and study of human induced pluripotent stem cells. Cell Stem Cell.

[20] Merkl C., Saalfrank A., Riesen N., Kühn R., Pertek A., Eser S., Hardt M.S., Kind A., Saur D., Wurst W. (2013). Efficient generation of rat induced pluripotent stem cells using a non-viral inducible vector. PLoS ONE.

[21] Ichida J.K., Blanchard J., Lam K., Son E.Y., Chung J.E., Egli D., Loh K.M., Carter A.C., di Giorgio F.P., Koszka K. (2009). A small-molecule inhibitor of TGF-β signaling replaces Sox2 in reprogramming by inducing nanog. Cell Stem Cell.

[22] Lyssiotis C.A., Foreman R.K., Staerk J., Garcia M., Mathur D., Markoulaki S., Hanna J., Lairson L.L., Charette B.D., Bouchez L.C. (2009). Reprogramming of murine fibroblasts to induced pluripotent stem cells with chemical complementation of Klf4. Proc. Natl. Acad. Sci. USA.

[23] Lin T., Ambasudhan R., Yuan X., Li W., Hilcove S., Abujarour R., Lin X., Hahm H.S., Hao E., Hayek A. (2009). A chemical platform for improved induction of human iPSCs. Nat. Methods.

[24] Zhu S., Li W., Zhou H., Wei W., Ambasudhan R., Lin T., Kim J., Zhang K., Ding S. (2010). Reprogramming of human primary somatic cells by Oct4 and chemical compounds. Cell Stem Cell.

[25] Esteban M.A., Wang T., Qin B., Yang J., Qin D., Cai J., Li W., Weng Z., Chen J., Ni S. (2010). Vitamin C enhances the generation of mouse and human induced pluripotent stem cells. Cell Stem Cell.

[26] Shi Y., Desponts C., Do J.T., Hahm H.S., Schöler H.R., Ding S. (2008). Induction of pluripotent stem cells from mouse embryonic fibroblasts by Oct4 and Klf4 with small-molecule compounds. Cell Stem Cell.

[27] Huangfu D., Osafune K., Maehr R., Guo W., Eijkelenboom A., Chen S., Muhlestein W., Melton D.A. (2008). Induction of pluripotent stem cells from primary human fibroblasts with only Oct4 and Sox2. Nat. Biotechnol..

[28] Lin T., Wu S. (2015). Reprogramming with small molecules instead of exogenous transcription factors. Stem Cells Int..

[29] Hou P., Li Y., Zhang X., Liu C., Guan J., Li H., Zhao T., Ye J., Yang W., Liu K. (2013). Pluripotent stem cells induced from mouse somatic cells by small-molecule compounds. Science.

[30] Okita K., Nakagawa M., Hyenjong H., Ichisaka T., Yamanaka S. (2008). Generation of mouse induced pluripotent stem cells without viral vectors. Science.

[31] Yu J., Hu K., Smuga-Otto K., Tian S., Stewart R., Slukvin I.I., Thomson J.A. (2009). Human induced pluripotent stem cells free of vector and transgene sequences. Science.

[32] Fusaki N., Ban H., Nishiyama A., Saeki K., Hasegawa M. (2009). Efficient induction of transgene-free human pluripotent stem cells using a vector based on Sendai virus, an RNA virus that does not integrate into the host genome. Proc. Jpn. Acad. Ser. B Phys. Biol. Sci..

[33] Seki T., Yuasa S., Oda M., Egashira T., Yae K., Kusumoto D., Nakata H., Tohyama S., Hashimoto H., Kodaira M. (2010). Generation of induced pluripotent stem cells from human terminally differentiated circulating T cells. Cell Stem Cell.

[34] Stadtfeld M., Nagaya M., Utikal J., Weir G., Hochedlinger K. (2008). Induced pluripotent stem cells generated without viral integration. Science.

[35] Zhou W., Freed C.R. (2009). Adenoviral gene delivery can reprogram human fibroblasts to induced pluripotent stem cells. Stem Cells Dayt. Ohio.

[36] Chang C.W., Lai Y.S., Pawlik K.M., Liu K., Sun C.W., Li C., Schoeb T.R., Townes T.M. (2009). Polycistronic lentiviral vector for “hit and run” reprogramming of adult skin fibroblasts to induced pluripotent stem cells. Stem Cells Dayt. Ohio.

[37] Sommer A.G., Rozelle S.S., Sullivan S., Mills J.A., Park S.M., Smith B.W., Iyer A.M., French D.L., Kotton D.N., Gadue P. (2012). Generation of human induced pluripotent stem cells from peripheral blood using the STEMCCA lentiviral vector. J. Vis. Exp..

[38] Yusa K., Rad R., Takeda J., Bradley A. (2009). Generation of transgene-free induced pluripotent mouse stem cells by the piggyBAC transposon. Nat. Methods.

[39] Warren L., Manos P.D., Ahfeldt T., Loh Y.H., Li H., Lau F., Ebina W., Mandal P.K., Smith Z.D., Meissner A. (2010). Highly efficient reprogramming to pluripotency and directed differentiation of human cells with synthetic modified mRNA. Cell Stem Cell.

[40] Plews J.R., Li J., Jones M., Moore H.D., Mason C., Andrews P.W., Na J. (2010). Activation of pluripotency genes in human fibroblast cells by a novel mRNA based approach. PLoS ONE.

[41] Zhou H., Wu S., Joo J.Y., Zhu S., Han D.W., Lin T., Trauger S., Bien G., Yao S., Zhu Y. (2009). Generation of induced pluripotent stem cells using recombinant proteins. Cell Stem Cell.

[42] Kim D., Kim C.H., Moon J.I., Chung Y.G., Chang M.Y., Han B.S., Ko S., Yang E., Cha K.Y., Lanza R. (2009). Generation of human induced pluripotent stem cells by direct delivery of reprogramming proteins. Cell Stem Cell.

[43] Lee C.H., Kim J.H., Lee H.J., Jeon K., Lim H., Choi H., Lee E.R., Park S.H., Park J.Y., Hong S. (2011). The generation of iPS cells using non-viral magnetic nanoparticle based transfection. Biomaterials.

[44] Anokye-Danso F., Trivedi C.M., Juhr D., Gupta M., Cui Z., Tian Y., Zhang Y., Yang W., Gruber P.J., Epstein J.A. (2011). Highly efficient miRNA-mediated reprogramming of mouse and human somatic cells to pluripotency. Cell Stem Cell.

[45] Hanna J., Markoulaki S., Schorderet P., Carey B.W., Beard C., Wernig M., Creyghton M.P., Steine E.J., Cassady J.P., Foreman R. (2008). Direct reprogramming of terminally differentiated mature B lymphocytes to pluripotency. Cell.

[46] Eminli S., Utikal J., Arnold K., Jaenisch R., Hochedlinger K. (2008). Reprogramming of neural progenitor cells into induced pluripotent stem cells in the absence of exogenous Sox2 expression. Stem Cells Dayt. Ohio.

[47] Aoki T., Ohnishi H., Oda Y., Tadokoro M., Sasao M., Kato H., Hattori K., Ohgushi H. (2010). Generation of induced pluripotent stem cells from human adipose-derived stem cells without c-MYC. Tissue Eng. Part A.

[48] Aasen T., Raya A., Barrero M.J., Garreta E., Consiglio A., Gonzalez F., Vassena R., Bilić J., Pekarik V., Tiscornia G. (2008). Efficient and rapid generation of induced pluripotent stem cells from human keratinocytes. Nat. Biotechnol..

[49] Loh Y.H., Hartung O., Li H., Guo C., Sahalie J.M., Manos P.D., Urbach A., Heffner G.C., Grskovic M., Vigneault F. (2010). Reprogramming of T cells from human peripheral blood. Cell Stem Cell.

[50] Staerk J., Dawlaty M.M., Gao Q., Maetzel D., Hanna J., Sommer C.A., Mostoslavsky G., Jaenisch R. (2010). Reprogramming of human peripheral blood cells to induced pluripotent stem cells. Cell Stem Cell.

[51] Wang Y., Liu J., Tan X., Li G., Gao Y., Liu X., Zhang L., Li Y. (2013). Induced pluripotent stem cells from human hair follicle mesenchymal stem cells. Stem Cell Rev..

[52] Li Q., Fan Y., Sun X., Yu Y. (2013). Generation of induced pluripotent stem cells from human amniotic fluid cells by reprogramming with two factors in feeder-free conditions. J. Reprod. Dev..

[53] Ruiz S., Brennand K., Panopoulos A.D., Herrerías A., Gage F.H., Izpisua-Belmonte J.C. (2010). High-efficient generation of induced pluripotent stem cells from human astrocytes. PLoS ONE.

[54] Park I.H., Zhao R., West J.A., Yabuuchi A., Huo H., Ince T.A., Lerou P.H., Lensch M.W., Daley G.Q. (2008). Reprogramming of human somatic cells to pluripotency with defined factors. Nature.

[55] Bibikova M., Golic M., Golic K.G., Carroll D. (2002). Targeted chromosomal cleavage and mutagenesis in Drosophila using zinc-finger nucleases. Genetics.

[56] Perez E.E., Wang J., Miller J.C., Jouvenot Y., Kim K.A., Liu O., Wang N., Lee G., Bartsevich V.V., Lee Y.L. (2008). Establishment of HIV-1 resistance in CD^4+^ T cells by genome editing using zinc-finger nucleases. Nat. Biotechnol..

[57] Bibikova M., Carroll D., Segal D.J., Trautman J.K., Smith J., Kim Y.G., Chandrasegaran S. (2001). Stimulation of homologous recombination through targeted cleavage by chimeric nucleases. Mol. Cell. Biol..

[58] Rong Z., Zhu S., Xu Y., Fu X. (2014). Homologous recombination in human embryonic stem cells using CRISPR/Cas9 nickase and a long DNA donor template. Protein Cell.

[59] Smith J., Grizot S., Arnould S., Duclert A., Epinat J.C., Chames P., Prieto J., Redondo P., Blanco F.J., Bravo J. (2006). A combinatorial approach to create artificial homing endonucleases cleaving chosen sequences. Nucleic Acids Res..

[60] Liu J., Stormo G.D. (2008). Context-dependent DNA recognition code for C_2_H_2_ zinc-finger transcription factors. Bioinform. Oxf. Engl..

[61] Durai S., Mani M., Kandavelou K., Wu J., Porteus M.H., Chandrasegaran S. (2005). Zinc finger nucleases: Custom-designed molecular scissors for genome engineering of plant and mammalian cells. Nucleic Acids Res..

[62] Li T., Yang B. (2013). TAL effector nuclease (TALEN) engineering. Methods Mol. Biol..

[63] Gogarten J.P., Hilario E. (2006). Inteins, introns, and homing endonucleases: Recent revelations about the life cycle of parasitic genetic elements. BMC Evol. Biol..

[64] Cox D.B.T., Platt R.J., Zhang F. (2015). Therapeutic genome editing: Prospects and challenges. Nat. Med..

[65] Silva G., Poirot L., Galetto R., Smith J., Montoya G., Duchateau P., Paques F. (2011). Meganucleases and other tools for targeted genome engineering: Perspectives and challenges for gene therapy. Curr. Gene Ther..

[66] Cathomen T., Joung J.K. (2008). Zinc-finger nucleases: The next generation emerges. Mol. Ther. J. Am. Soc. Gene Ther..

[67] Kim Y.G., Cha J., Chandrasegaran S. (1996). Hybrid restriction enzymes: Zinc finger fusions to Fok I cleavage domain. Proc. Natl. Acad. Sci. USA.

[68] Ramirez C.L., Foley J.E., Wright D.A., Müller-Lerch F., Rahman S.H., Cornu T.I., Winfrey R.J., Sander J.D., Fu F., Townsend J.A. (2008). Unexpected failure rates for modular assembly of engineered zinc fingers. Nat. Methods.

[69] Christian M., Cermak T., Doyle E.L., Schmidt C., Zhang F., Hummel A., Bogdanove A.J., Voytas D.F. (2010). Targeting DNA double-strand breaks with TAL effector nucleases. Genetics.

[70] Reyon D., Tsai S.Q., Khayter C., Foden J.A., Sander J.D., Joung J.K. (2012). FLASH assembly of TALENs for high-throughput genome editing. Nat. Biotechnol..

[71] Carlson D.F., Fahrenkrug S.C., Hackett P.B. (2012). Targeting DNA with fingers and TALENs. Mol. Ther. Nucleic Acids.

[72] Barrangou R., Fremaux C., Deveau H., Richards M., Boyaval P., Moineau S., Romero D.A., Horvath P. (2007). CRISPR provides acquired resistance against viruses in prokaryotes. Science.

[73] Wang H., Yang H., Shivalila C.S., Dawlaty M.M., Cheng A.W., Zhang F., Jaenisch R. (2013). One-step generation of mice carrying mutations in multiple genes by CRISPR/Cas-mediated genome engineering. Cell.

[74] Jinek M., Chylinski K., Fonfara I., Hauer M., Doudna J.A., Charpentier E. (2012). A Programmable dual-RNA-guided DNA endonuclease in adaptive bacterial immunity. Science.

[75] Fu Y., Foden J.A., Khayter C., Maeder M.L., Reyon D., Joung J.K., Sander J.D. (2013). High-frequency off-target mutagenesis induced by CRISPR-Cas nucleases in human cells. Nat. Biotechnol..

[76] Ishino Y., Shinagawa H., Makino K., Amemura M., Nakata A. (1987). Nucleotide sequence of the *IAP* gene, responsible for alkaline phosphatase isozyme conversion in Escherichia coli, and identification of the gene product. J. Bacteriol..

[77] Mojica F.J.M., Díez-Villaseñor C., García-Martínez J., Soria E. (2005). Intervening sequences of regularly spaced prokaryotic repeats derive from foreign genetic elements. J. Mol. Evol..

[78] Mali P., Yang L., Esvelt K.M., Aach J., Guell M., di Carlo J.E., Norville J.E., Church G.M. (2013). RNA-guided human genome engineering via Cas9. Science.

[79] Garneau J.E., Dupuis M.È., Villion M., Romero D.A., Barrangou R., Boyaval P., Fremaux C., Horvath P., Magadán A.H., Moineau S. (2010). The CRISPR/Cas bacterial immune system cleaves bacteriophage and plasmid DNA. Nature.

[80] Ran F.A., Hsu P.D., Lin C.Y., Gootenberg J.S., Konermann S., Trevino A.E., Scott D.A., Inoue A., Matoba S., Zhang Y. (2013). Double nicking by RNA-guided CRISPR Cas9 for enhanced genome editing specificity. Cell.

[81] Mali P., Aach J., Stranges P.B., Esvelt K.M., Moosburner M., Kosuri S., Yang L., Church G.M. (2013). CAS9 transcriptional activators for target specificity screening and paired nickases for cooperative genome engineering. Nat. Biotechnol..

[82] Meyer M., Ortiz O., Hrabé de Angelis M., Wurst W., Kühn R. (2012). Modeling disease mutations by gene targeting in one-cell mouse embryos. Proc. Natl. Acad. Sci. USA.

[83] Soldner F., Laganiere J., Cheng A.W., Hockemeyer D., Gao Q., Alagappan R., Khurana V., Golbe L.I., Myers R.H., Lindquist S. (2011). Generation of isogenic pluripotent stem cells differing exclusively at two early onset Parkinson point mutations. Cell.

[84] Bellin M., Casini S., Davis R.P., Aniello C.D., Haas J., Ward-van Oostwaard D., Tertoolen L.G.J., Jung C.B., Elliott D.A., Welling A. (2013). Isogenic human pluripotent stem cell pairs reveal the role of a KCNH2 mutation in long-QT syndrome. EMBO J..

[85] Egashira T., Yuasa S., Fukuda K. (2013). Novel insights into disease modeling using induced pluripotent stem cells. Biol. Pharm. Bull..

[86] Egashira T., Yuasa S., Suzuki T., Aizawa Y., Yamakawa H., Matsuhashi T., Ohno Y., Tohyama S., Okata S., Seki T. (2012). Disease characterization using LQTS-specific induced pluripotent stem cells. Cardiovasc. Res..

[87] Shefner J.M., Cudkowicz M.E., Schoenfeld D., Conrad T., Taft J., Chilton M., Urbinelli L., Qureshi M., Zhang H., Pestronk A. (2004). NEALS Consortium. A clinical trial of creatine in ALS. Neurology.

[88] Singh B., Gupta R.S. (1985). Species-specific differences in the toxicity and mutagenicity of the anticancer drugs mithramycin, chromomycin A3, and olivomycin. Cancer Res..

[89] Chun Y.S. (2010). Applications of patient-specific induced pluripotent stem cells; focused on disease modeling, drug screening and therapeutic potentials for liver disease. Int. J. Biol. Sci..

[90] Gore A., Li Z., Fung H.L., Young J.E., Agarwal S., Antosiewicz-Bourget J., Canto I., Giorgetti A., Israel M.A., Kiskinis E. (2011). Somatic coding mutations in human induced pluripotent stem cells. Nature.

[91] Ohi Y., Qin H., Hong C., Blouin L., Polo J.M., Guo T., Qi Z., Downey S.L., Manos P.D., Rossi D.J. (2011). Incomplete DNA methylation underlies a transcriptional memory of somatic cells in human iPS cells. Nat. Cell Biol..

[92] Laurent L.C., Ulitsky I., Slavin I., Tran H., Schork A., Morey R., Lynch C., Harness J.V., Lee S., Barrero M.J. (2011). Dynamic changes in the copy number of pluripotency and cell proliferation genes in human ESCs and iPSCs during reprogramming and time in culture. Cell Stem Cell.

[93] Mekhoubad S., Bock C., de Boer A.S., Kiskinis E., Meissner A., Eggan K. (2012). Erosion of dosage compensation impacts human iPSC disease modeling. Cell Stem Cell.

[94] Moad M., Pal D., Hepburn A.C., Williamson S.C., Wilson L., Lako M., Armstrong L., Hayward S.W., Franco O.E., Cates J.M. (2013). A novel model of urinary tract differentiation, tissue regeneration, and disease: Reprogramming human prostate and bladder cells into induced pluripotent stem cells. Eur. Urol..

[95] Nguyen H.N., Byers B., Cord B., Shcheglovitov A., Byrne J., Gujar P., Kee K., Schüle B., Dolmetsch R.E., Langston W. (2011). *LRRK2* mutant iPSC-derived DA neurons demonstrate increased susceptibility to oxidative stress. Cell Stem Cell.

[96] Israel M.A., Yuan S.H., Bardy C., Reyna S.M., Mu Y., Herrera C., Hefferan M.P., van Gorp S., Nazor K.L., Boscolo F.S. (2012). Probing sporadic and familial Alzheimer’s disease using induced pluripotent stem cells. Nature.

[97] Dimos J.T., Rodolfa K.T., Niakan K.K., Weisenthal L.M., Mitsumoto H., Chung W., Croft G.F., Saphier G., Leibel R., Goland R. (2008). Induced pluripotent stem cells generated from patients with ALS can be differentiated into motor neurons. Science.

[98] Ebert A.D., Yu J., Rose F.F., Mattis V.B., Lorson C.L., Thomson J.A., Svendsen C.N. (2009). Induced pluripotent stem cells from a spinal muscular atrophy patient. Nature.

[99] Juopperi T.A., Kim W.R., Chiang C.H., Yu H., Margolis R.L., Ross C.A., Ming G., Song H. (2012). Astrocytes generated from patient induced pluripotent stem cells recapitulate features of Huntington’s disease patient cells. Mol. Brain.

[100] Sheridan S.D., Theriault K.M., Reis S.A., Zhou F., Madison J.M., Daheron L., Loring J.F., Haggarty S.J. (2011). Epigenetic characterization of the *FMR1* gene and aberrant neurodevelopment in human induced pluripotent stem cell models of fragile X syndrome. PLoS ONE.

[101] Wang X.M., Yik W.Y., Zhang P., Lu W., Dranchak P.K., Shibata D., Steinberg S.J., Hacia J.G. (2012). The gene expression profiles of induced pluripotent stem cells from individuals with childhood cerebral adrenoleukodystrophy are consistent with proposed mechanisms of pathogenesis. Stem Cell Res. Ther..

[102] Lu H.E., Yang Y.C., Chen S.M., Su H.L., Huang P.C., Tsai M.S., Wang T.H., Tseng C.P., Hwang S.M. (2013). Modeling neurogenesis impairment in Down syndrome with induced pluripotent stem cells from Trisomy 21 amniotic fluid cells. Exp. Cell Res..

[103] Batista L.F.Z., Pech M.F., Zhong F.L., Nguyen H.N., Xie K.T., Zaug A.J., Crary S.M., Choi J., Sebastiano V., Cherry A. (2011). Telomere shortening and loss of self-renewal in dyskeratosis congenita induced pluripotent stem cells. Nature.

[104] Ku S., Soragni E., Campau E., Thomas E.A., Altun G., Laurent L.C., Loring J.F., Napierala M., Gottesfeld J.M. (2010). Friedreich’s ataxia induced pluripotent stem cells model intergenerational GAATTC triplet repeat instability. Cell Stem Cell.

[105] Fujikura J., Nakao K., Sone M., Noguchi M., Mori E., Naito M., Taura D., Harada-Shiba M., Kishimoto I., Watanabe A. (2012). Induced pluripotent stem cells generated from diabetic patients with mitochondrial DNA A3243G mutation. Diabetologia.

[106] Jiang Y., Cowley S.A., Siler U., Melguizo D., Tilgner K., Browne C., Dewilton A., Przyborski S., Saretzki G., James W.S. (2012). Derivation and functional analysis of patient-specific induced pluripotent stem cells as an *in vitro* model of chronic granulomatous disease. Stem Cells Dayt. Ohio.

[107] Kumano K., Arai S., Hosoi M., Taoka K., Takayama N., Otsu M., Nagae G., Ueda K., Nakazaki K., Kamikubo Y. (2012). Generation of induced pluripotent stem cells from primary chronic myelogenous leukemia patient samples. Blood.

[108] Müller L.U.W., Schlaeger T.M., de Vine A.L., Williams D.A. (2012). Induced pluripotent stem cells as a tool for gaining new insights into Fanconi anemia. Cell Cycle Georget. Tex.

[109] Rashid S.T., Corbineau S., Hannan N., Marciniak S.J., Miranda E., Alexander G., Huang-Doran I., Griffin J., Ahrlund-Richter L., Skepper J. (2010). Modeling inherited metabolic disorders of the liver using human induced pluripotent stem cells. J. Clin. Investig..

[110] Yang J., Cai J., Zhang Y., Wang X., Li W., Xu J., Li F., Guo X., Deng K., Zhong M. (2010). Induced pluripotent stem cells can be used to model the genomic imprinting disorder Prader-Willi syndrome. J. Biol. Chem..

[111] Chamberlain S.J., Chen P.F., Ng K.Y., Bourgois-Rocha F., Lemtiri-Chlieh F., Levine E.S., Lalande M. (2010). Induced pluripotent stem cell models of the genomic imprinting disorders Angelman and Prader-Willi syndromes. Proc. Natl. Acad. Sci. USA.

[112] Moretti A., Bellin M., Welling A., Jung C.B., Lam J.T., Bott-Flügel L., Dorn T., Goedel A., Höhnke C., Hofmann F. (2010). Patient-specific induced pluripotent stem-cell models for long-QT syndrome. N. Engl. J. Med..

[113] Itzhaki I., Maizels L., Huber I., Zwi-Dantsis L., Caspi O., Winterstern A., Feldman O., Gepstein A., Arbel G., Hammerman H. (2011). Modelling the long QT syndrome with induced pluripotent stem cells. Nature.

[114] Yazawa M., Hsueh B., Jia X., Pasca A.M., Bernstein J.A., Hallmayer J., Dolmetsch R.E. (2011). Using induced pluripotent stem cells to investigate cardiac phenotypes in Timothy syndrome. Nature.

[115] Carvajal-Vergara X., Sevilla A., Souza S.L.D., Ang Y.S., Schaniel C., Lee D.F., Yang L., Kaplan A.D., Adler E.D., Rozov R. (2010). Patient-specific induced pluripotent stem-cell-derived models of LEOPARD syndrome. Nature.

[116] Zhang J., Lian Q., Zhu G., Zhou F., Sui L., Tan C., Mutalif R.A., Navasankari R., Zhang Y., Tse H.F. (2011). A human iPSC model of Hutchinson Gilford Progeria reveals vascular smooth muscle and mesenchymal stem cell defects. Cell Stem Cell.

[117] Itzhaki I., Maizels L., Huber I., Gepstein A., Arbel G., Caspi O., Miller L., Belhassen B., Nof E., Glikson M. (2012). Modeling of catecholaminergic polymorphic ventricular tachycardia with patient-specific human-induced pluripotent stem cells. J. Am. Coll. Cardiol..

[118] Sun N., Yazawa M., Liu J., Han L., Sanchez-Freire V., Abilez O.J., Navarrete E.G., Hu S., Wang L., Lee A. (2012). Patient-specific induced pluripotent stem cells as a model for familial dilated cardiomyopathy. Sci. Transl. Med..

[119] Kinnear C., Chang W.Y., Khattak S., Hinek A., Thompson T., de Carvalho Rodrigues D., Kennedy K., Mahmut N., Pasceri P., Stanford W.L. (2013). Modeling and rescue of the vascular phenotype of Williams-Beuren syndrome in patient induced pluripotent stem cells. Stem Cells Transl. Med..

[120] Jin Z.B., Okamoto S., Osakada F., Homma K., Assawachananont J., Hirami Y., Iwata T., Takahashi M. (2011). Modeling retinal degeneration using patient-specific induced pluripotent stem cells. PLoS ONE.

[121] Chen Y., Luo R., Xu Y., Cai X., Li W., Tan K., Huang J., Dai Y. (2013). Generation of systemic lupus erythematosus-specific induced pluripotent stem cells from urine. Rheumatol. Int..

[122] Saitta B., Passarini J., Sareen D., Ornelas L., Sahabian A., Argade S., Krakow D., Cohn D.H., Svendsen C.N., Rimoin D.L. (2014). Patient-derived skeletal dysplasia induced pluripotent stem cells display abnormal chondrogenic marker expression and regulation by BMP2 and TGFβ1. Stem Cells Dev..

[123] Lee D.F., Su J., Kim H.S., Chang B., Papatsenko D., Zhao R., Yuan Y., Gingold J., Xia W., Darr H. (2015). Modeling familial cancer with induced pluripotent stem cells. Cell.

[124] Singh V.K., Kalsan M., Kumar N., Saini A., Chandra R. (2015). Induced pluripotent stem cells: Applications in regenerative medicine, disease modeling, and drug discovery. Front. Cell Dev. Biol..

[125] Ghodsizadeh A., Taei A., Totonchi M., Seifinejad A., Gourabi H., Pournasr B., Aghdami N., Malekzadeh R., Almadani N., Salekdeh G.H. (2010). Generation of liver disease-specific induced pluripotent stem cells along with efficient differentiation to functional hepatocyte-like cells. Stem Cell Rev..

[126] Choi S.M., Kim Y., Shim J.S., Park J.T., Wang R.H., Leach S.D., Liu J.O., Deng C., Ye Z., Jang Y.Y. (2013). Efficient drug screening and gene correction for treating liver disease using patient-specific stem cells. Hepatology.

[127] Irudayam J.I. (2014). Modeling Liver Diseases Using Induced Pluripotent Stem Cell (Ipsc)-Derived Hepatocytes. J. Stem Cell Res. Ther..

[128] Ye Z., Zhan H., Mali P., Dowey S., Williams D.M., Jang Y.Y., Dang C.V., Spivak J.L., Moliterno A.R., Cheng L. (2009). Human-induced pluripotent stem cells from blood cells of healthy donors and patients with acquired blood disorders. Blood.

[129] Trevisan M., Sinigaglia A., Desole G., Berto A., Pacenti M., Palù G., Barzon L. (2015). Modeling Viral Infectious Diseases and Development of Antiviral Therapies Using Human Induced Pluripotent Stem Cell-Derived Systems. Viruses.

[130] Lafaille F.G., Pessach I.M., Zhang S.Y., Ciancanelli M.J., Herman M., Abhyankar A., Ying S.W., Keros S., Goldstein P.A., Mostoslavsky G. (2012). Impaired intrinsic immunity to HSV-1 in human iPSC-derived TLR3-deficient CNS cells. Nature.

[131] Ciancanelli M.J., Huang S.X.L., Luthra P., Garner H., Itan Y., Volpi S., Lafaille F.G., Trouillet C., Schmolke M., Albrecht R.A. (2015). Infectious disease. Life-threatening influenza and impaired interferon amplification in human IRF7 deficiency. Science.

[132] Kambal A., Mitchell G., Cary W., Gruenloh W., Jung Y., Kalomoiris S., Nacey C., McGee J., Lindsey M., Fury B. (2011). Generation of HIV-1 resistant and functional macrophages from hematopoietic stem cell-derived induced pluripotent stem cells. Mol. Ther. J. Am. Soc. Gene Ther..

[133] Kaur G., Dufour J.M. (2012). Cell lines: Valuable tools or useless artifacts. Spermatogenesis.

[134] Kim J., Hoffman J.P., Alpaugh R.K., Rhim A.D., Rhimm A.D., Reichert M., Stanger B.Z., Furth E.E., Sepulveda A.R., Yuan C.X. (2013). An iPSC line from human pancreatic ductal adenocarcinoma undergoes early to invasive stages of pancreatic cancer progression. Cell Rep..

[135] Riggs J.W., Barrilleaux B.L., Varlakhanova N., Bush K.M., Chan V., Knoepfler P.S. (2013). Induced pluripotency and oncogenic transformation are related processes. Stem Cells Dev..

[136] Curry E.L., Moad M., Robson C.N., Heer R. (2015). Using induced pluripotent stem cells as a tool for modelling carcinogenesis. World J. Stem Cells.

[137] Wong D.J., Liu H., Ridky T.W., Cassarino D., Segal E., Chang H.Y. (2008). Module map of stem cell genes guides creation of epithelial cancer stem cells. Cell Stem Cell.

[138] Kim K., Doi A., Wen B., Ng K., Zhao R., Cahan P., Kim J., Aryee M.J., Ji H., Ehrlich L.I.R. (2010). Epigenetic memory in induced pluripotent stem cells. Nature.

[139] Osafune K., Caron L., Borowiak M., Martinez R.J., Fitz-Gerald C.S., Sato Y., Cowan C.A., Chien K.R., Melton D.A. (2008). Marked differences in differentiation propensity among human embryonic stem cell lines. Nat. Biotechnol..

[140] Soldner F., Jaenisch R. (2012). iPSC disease modeling. Science.

[141] Soldner F., Hockemeyer D., Beard C., Gao Q., Bell G.W., Cook E.G., Hargus G., Blak A., Cooper O., Mitalipova M. (2009). Parkinson’s disease patient-derived induced pluripotent stem cells free of viral reprogramming factors. Cell.

[142] Meissner A. (2010). Epigenetic modifications in pluripotent and differentiated cells. Nat. Biotechnol..

[143] Takebe T., Sekine K., Enomura M., Koike H., Kimura M., Ogaeri T., Zhang R.R., Ueno Y., Zheng Y.W., Koike N. (2013). Vascularized and functional human liver from an iPSC-derived organ bud transplant. Nature.

[144] Lancaster M.A., Renner M., Martin C.A., Wenzel D., Bicknell L.S., Hurles M.E., Homfray T., Penninger J.M., Jackson A.P., Knoblich J.A. (2013). Cerebral organoids model human brain development and microcephaly. Nature.

[145] Zhang D., Pekkanen-Mattila M., Shahsavani M., Falk A., Teixeira A.I., Herland A. (2014). A 3D Alzheimer’s disease culture model and the induction of P21-activated kinase mediated sensing in iPSC derived neurons. Biomaterials.

[146] Eiraku M., Takata N., Ishibashi H., Kawada M., Sakakura E., Okuda S., Sekiguchi K., Adachi T., Sasai Y. (2011). Self-organizing optic-cup morphogenesis in three-dimensional culture. Nature.

[147] Xia Y., Sancho-Martinez I., Nivet E., Rodriguez Esteban C., Campistol J.M., Izpisua Belmonte J.C. (2014). The generation of kidney organoids by differentiation of human pluripotent cells to ureteric bud progenitor-like cells. Nat. Protoc..

[148] Miller J.D., Ganat Y.M., Kishinevsky S., Bowman R.L., Liu B., Tu E.Y., Mandal P.K., Vera E., Shim J., Kriks S. (2013). Human iPSC-based modeling of late-onset disease via progerin-induced aging. Cell Stem Cell.

[149] Koch P., Breuer P., Peitz M., Jungverdorben J., Kesavan J., Poppe D., Doerr J., Ladewig J., Mertens J., Tüting T. (2011). Excitation-induced ataxin-3 aggregation in neurons from patients with Machado-Joseph disease. Nature.

